# Particle Size, Surface Area, and Amorphous Content as Predictors of Solubility and Bioavailability for Five Commercial Sources of Ferric Orthophosphate in Ready-To-Eat Cereal

**DOI:** 10.3390/nu8030129

**Published:** 2016-03-01

**Authors:** Robin S. Dickmann, Gale M. Strasburg, Dale R. Romsos, Lori A. Wilson, Grace H. Lai, Hsimin Huang

**Affiliations:** 1 Kellogg Company, W.K. Kellogg Institute for Food and Nutrition Research, Battle Creek, 2 Hamblin Avenue, Battle Creek, MI 49015, USA; lori.wilson@kellogg.com (L.A.W.); gracehlai@hotmail.com (G.H.L.); hsimin.huang@kellogg.com (H.H.); 2 Department of Food Science and Human Nutrition, Michigan State University, 469 Wilson Road, East Lansing, MI 48824, USA; stragale@anr.msu.edu (G.M.S.); dromsos@anr.msu.edu (D.R.R.)

**Keywords:** ferric orthophosphate, reduced iron, bioavailability, solubility, particle size, surface area, amorphous content, X-ray diffraction, dynamic vapor sorption, physicochemical properties

## Abstract

Ferric orthophosphate (FePO_4_) has had limited use as an iron fortificant in ready-to-eat (RTE) cereal because of its variable bioavailability, the mechanism of which is poorly understood. Even though FePO_4_ has desirable sensory properties as compared to other affordable iron fortificants, few published studies have well-characterized its physicochemical properties. Semi-crystalline materials such as FePO_4_ have varying degrees of molecular disorder, referred to as amorphous content, which is hypothesized to be an important factor in bioavailability. The objective of this study was to systematically measure the physicochemical factors of particle size, surface area, amorphous content, and solubility underlying the variation in FePO_4_ bioavailability. Five commercial FePO_4_ sources and ferrous sulfate were added to individual batches of RTE cereal. The relative bioavailability value (RBV) of each iron source, determined using the AOAC Rat Hemoglobin Repletion Bioassay, ranged from 51% to 99% (*p* < 0.05), which is higher than typically reported. Solubility in dilute HCl accurately predicted RBV (*R^2^* = 0.93, *p* = 0.008). Amorphous content measured by Dynamic Vapor Sorption ranged from 1.7% to 23.8% and was a better determinant of solubility (*R^2^* = 0.91; *p* = 0.0002) than surface area (*R^2^* = 0.83; *p* = 0.002) and median particle size (*R^2^* = 0.59; *p* = 0.12). The results indicate that while solubility of FePO_4_ is highly predictive of RBV, solubility, in turn, is strongly linked to amorphous content and surface area. This information may prove useful for the production of FePO_4_ with the desired RBV.

## 1. Introduction

Fortifying foods with inorganic iron remains a challenge. Many forms of inorganic iron can be used to fortify food but few forms provide the desired combination of bioavailability and product stability. Freely soluble iron forms such as ferrous sulfate have the highest bioavailability since iron and must be solubilized in gastric juice before it can be absorbed by the body [1,2,3,4]. However, soluble iron is also a pro-oxidant, has a metallic off-taste and reacts with many food components causing negative organoleptic changes and shortened shelf life [5,6,7]. As a result, less soluble and thus less reactive forms of iron are often chosen for food applications. To be absorbed by the small intestine, some portion of the dietary iron must first dissolve in stomach acid [4]. The solubilities of the various elemental or compound forms of iron depend on their method of manufacture which, in turn, may result in a wide range of bioavailabilities even for the same form of iron [3,5,7,8,9]. Concerns were raised by the scientific community at the Monterrey Workshop in 2000 about the efficacy of elemental iron due to its variable and often poor bioavailability reported in the literature [2]. Nevertheless, elemental iron is the most widely used iron fortificant in cereal and cereal products due to its low cost and fewer stability and organoleptic problems [10]. Results of the SUSTAIN Task Force on Iron Powders collaborative study found the RBVs of commercial elemental iron powders to vary considerably (RBV 21%–64%, relative to ferrous sulfate, defined as RBV 100%) [10,11]. Commonly used, affordable forms of iron with excellent bioavailability include for example, ferrous sulfate and ferrous fumarate [1,3,12]. However, these highly bioavailable forms of iron pose sensory and stability issues due to the accompanying increase in iron solubility within the food matrix.

Ferric orthophosphate (FePO_4_) is preferred over elemental iron for liquid products, light- colored food, and oxidatively sensitive food applications because of its low density, light color, good stability, and non-metallic flavor. However, historically FePO_4_ is thought to have little nutritional value and this has limited its use as a fortificant [1,7,13,14]. According to a Mintel Global New Product Database (GNPD) search from January 1996–January 2016, there were 2121 food and beverage products including snack/cereal/energy bars, hot and cold cereals, enriched rice and meal replacement drinks containing FePO_4_ marketed globally. This compares to over 18,900 food and beverage products marketed during the same period that were fortified with all types of iron [15]. There is limited information in the literature about the physicochemical properties of FePO_4_ and their influence on bioavailability; the paucity of information preclude its widespread use as a food fortificant. Therefore, the objectives of this study were to characterize the physicochemical properties of five sources of commercially available, food-grade FePO_4_ to determine which properties have the greatest influence on *in vivo* relative bioavailability (RBV). Solubility, particle size, surface area, and amorphous content of each of the FePO_4_ powders were quantified and correlated with RBV.

## 2. Experimental Section

### 2.1. Iron Sources

FePO_4_ Sources 1, 2, 3, and 4 were obtained from Budenheim Chemische Fabrik (Mainz, Germany) and had product numbers 53–80, 53–81, 53–82, and 53–85, respectively. FePO_4_ Source 5 was obtained from Madison Chemicals, Inc. (Madison Township, NJ, USA). By supplier specification, all FePO_4_ sources were food grade and >99% pure; pH ranged from 3 to 5. Standard ferrous sulfate heptahydrate (Source 6) was purchased from Mallinckrodt Baker, Inc. (Phillipsburg, NJ, USA). The color and texture of the FePO_4_ powders varied. Source 1 was a dull-yellow, fine powder that clung to surfaces, Sources 2, 3, and 4 were beige to off-white powders with poor flow properties, and Source 5 was a pinkish-white, talc-like powder with good flowability. There were also distinct differences in organoleptic characteristics among the FePO_4_ powders, with Source 1 exhibiting an objectionable strong metallic taste and odor and Source 5 exhibiting a desirable bland flavor. Supplier information stated that the products varied in particle size and therefore solubility in water and dilute mineral acids.

Physical property measurements were made on the same lots of FePO_4_ that were used for the AOAC Rat Hemoglobin Repletion Study, identified as lot 1 for Source 1–5. Additional production lots were available for Source 1 (one lot), Source 2 (one lot, particle size and surface area only), and Source 3 (two lots) and analyzed to indicate lot-to-lot process variation.

### 2.2. AOAC Rat Hemoglobin Repletion Bioassay

Relative bioavailability value (RBV) of the FePO_4_ was determined using the AOAC Rat Hemoglobin Repletion Bioassay/slope ratio method [16]. Iron-deficient rats were given repletion diets fortified with graded quantities of FePO_4_ powders, ferrous sulfate, or a no-added-iron diet. The study was approved by the Institutional Animal Care and Use Committee at Covance Laboratory in Madison, Wisconsin and an exemption was granted by the Michigan State University Institutional Animal Care and Use Committee (IRB#: X08-521).

#### 2.2.1. Animals

Male Hsd: Sprague Dawley^®^ SD^®^ rats were procured at 21 days of age from Harlan Sprague Dawley, Inc. (Madison, WI, USA). They were single-housed in wire-bottomed, stainless steel cages and subjected to a 12-h light/12-h dark cycle. Upon arrival, the rats were immediately fed ad libitum the iron-depletion diet for 15 days. Blood was collected from the jugular vein at the end of the iron-depletion period and analyzed for hemoglobin concentration. The animals were then randomly assigned to test diet groups at 36 days of age with 10 animals per group. The animals were fed ad libitum their respective iron-repletion diets for two weeks from clear glass jars. Fresh food was offered on day 1 and day 8. Individual animal body weight data were recorded on days 1, 8, and 15, and individual food consumption data were recorded weekly during the test period. The animals were bled on day 15 and the final hemoglobin levels determined.

#### 2.2.2. Animal Iron-Depletion and Repletion Diets

The iron-depletion animal diet, Diet TD 0396, was purchased from Harlan Teklad (Madison, WI, USA), and included 35.0 g/kg iron-deficient mineral mix (TD81062) and 10.0 g/kg vitamin mix AIN-76A (TD40077). The animals also received ad libitum a deionized water source (analyzed for iron content).

Seven batches of RTE-cereal were produced and used to make the rat iron repletion diets. Five batches were made with the five respective FePO_4_ sources, a sixth batch was made with ferrous sulfate, and a seventh batch without any added iron. Ingredients in the RTE-cereal included whole corn grits, malted barley flavor, corn syrup, sugar, salt, and the following added vitamins per 30 g serving of finished product: A (500 IU), C (6 mg), D (40 IU), B-12 (1.5 μg), B-1 (0.4 mg), B-2 (0.4 mg), B-6 (0.5 mg), niacin (5 mg), and folate (0.1 mg). Each iron source was added to a 90-kg batch of corn cereal before processing to provide 8 mg of iron per 30 g cereal. The cereal, iron sources, and other added ingredients underwent a pressurized batch-cooking process (Kellogg Company pilot plant, Battle Creek, MI, USA) and then were tempered, flaked, and toasted. After processing, the diets were prepared in 1-kg batches to contain 60% (600 g) iron-depletion animal diet and 40% (400 g) RTE-cereal. The RTE-cereal made with and without added iron was proportioned to formulate target doses of 0, 6, 12, 24, or 48 mg of each test iron source per kg rat iron-repletion diet. The iron content of each iron source and test diet was measured using Inductively Coupled Plasma (ICP) mass spectrometry [17].

### 2.3. In Vitro Solubility

The method used for determination of the solubility of the iron sources was based on the method of Shah and others[6] and later modified by Forbes and others [18]. To decrease assay variability, the method was further modified to incorporate the dissolution apparatus and precisely controlled temperature, mixing, and filtering conditions as described in the United States Pharmacopeial Convention (USPC) 2006 Official Method for Dietary Supplements, #2040 Disintegration and Dissolution of Dietary Vitamin and Mineral Supplements. Approximately 0.29 mg total iron was analyzed per sample (*ca.* 1.12 g FePO_4_). Samples were dissolved in 450 mL (2–3 mg iron/mL) of one of the three following standardized HCl (Fisher Scientific, Pittsburg, PA, USA) solutions: 0.02 N, 0.05 N, or 0.10 N. The amount of dissolved iron was determined by ICP mass spectrometry and expressed as a percentage of iron dissolved (weight basis) [17].

### 2.4. Particle Size Distributions

The particle size distributions were determined by Particle Technology Labs, Ltd. (Method MM324.01) (Downers Grove, IL, USA) using a Malvern Mastersizer 2000 Laser Diffractor. Four 12-s analyses were made at one-minute intervals, and results reported as the average of the four analyses for each sample. The analysis was repeated six times for each lot of FePO_4_. The following four mean volume statistics were calculated: mean particle size diameter (μm); and three cumulative percent statistics D_10_, D_50_ (median), and D_90_ that are the percentages of the total sample less than the measured particle size (μm). The refractive indices were determined for the FePO_4_ samples by the Becke Line Test for the sodium line (589 nm) using the microscopic emersion method.

### 2.5. Surface Area

Surface area measurements were made by Particle Technology, Ltd. using a five-point Brunauer, Emmett, and Teller (B.E.T.) surface area analysis performed on a Quantachrome Autosorb-AS-1 Static-Pressure instrument (Method AU225.01). Samples of 2.2–2.6 g were weighed to four decimal places and prepared for analysis by out-gassing under a helium purge at 25 °C for 16 h until the samples were thoroughly dry and free of surface contamination. Analysis was carried out using nitrogen as the adsorbate gas (maintained at a temperature of 77.4 °K) over the following five B.E.T. points: 0.10, 0.15, 0.20, 0.25, and 0.30. The analysis was repeated six times for each lot of FePO_4_.

### 2.6. Amorphous Content

The microstructures of the FePO_4_ sources 1–5 were examined by polarized light microscopy using white light through crossed polarizers and first order red compensation in a light red background. FePO_4_ crystals are anisotropic (non-uniform) with two refractive indices (birefringence) when plane polarized light is passed through the material. The birefringent crystals appear brightly colored while the amorphous FePO_4_, lacking the molecular order and pattern of crystals, is not birefringent and appears clear.

Powder X-ray diffraction was performed by McCrone Associates, Inc. (Westmont, IL, USA) to profile the crystal structures of the FePO_4_ sources. The instrument used was a Siemens D5000 powder diffractometer. The samples were prepared as random powder specimens by loading into plastic dishes. The samples were X-rayed from 2 to 50 degrees 2-theta using copper radiation, an accelerating voltage of 40 kV/30 mA, a step size of 0.05 degrees, and a data acquisition time of 2.0 s per step, with the sample spinning.

The amorphous content of the FePO_4_ sources was determined by measuring the moisture uptake of the material by dynamic vapor sorption (DVS). The DVS moisture uptake method was developed by J.Y.Y. Heng and D.R. Williams (Department of Chemical Engineering, Imperial College, London, UK). A DVS-1 instrument (Surface Measurement Systems UK, Ltd., Alperton London, UK) equipped with a Cahn D-100 microbalance (sensitivity 0.1 μg) was used to produce isotherms for each of the FePO_4_ sources. Isotherm analyses were conducted by weighing *ca.* 50 mg of FePO_4_ into the microbalance module and pre-conditioning at 0% relative humidity (RH) for 300 min. Following preconditioning, the relative humidity (RH) was incrementally increased on average every 60 min as follows: 0, 10%, 20%, 30%, 40%, 50%, 60%, 70%, 80%, and 90% RH and then reversed to complete one sorption/desorption isotherm cycle over approximately 2000 min. A minimum of two consecutive isotherm cycles were conducted on each source of FePO_4_ to determine if the samples displayed moisture induced phase changes and to determine optimum cycle-time. Samples were held at each change in humidity until the sample weight (moisture uptake) stabilized.

A 100% amorphous FePO_4_ standard was prepared from Source 4. Source 4 was chosen because it was highly amorphous by X-ray diffraction and polarized light microscopy. The standard was prepared by completely dissolving FePO_4_ Source 4 in 0.10N HCl and spray drying (Buchi B-290 apparatus, Buchi, Oldham, UK) the material to form an amorphous powder. The amorphous content of each FePO_4_ source (1–5) and the standard were measured by DVS using the same isotherm analysis parameters previously described. The amorphous FePO_4_ standard material was extremely hygroscopic and mass equilibration was closely approximated but not fully reached above 10% RH during the 2000-min isotherm cycle-time. Therefore, estimations of amorphous content were made by a ratio comparison of the moisture uptake of each FePO_4_ source to the 100% amorphous standard using the data at 10% RH.

### 2.7. Statistical Analysis

RBVs were based on the change in hemoglobin concentration (g/L) from the end of the iron-depletion period to the end of the iron-repletion period (day-15). Hemoglobin values from rats on FePO_4_ test diets 1–5 were compared to that of the standard ferrous sulfate treatment (Diet 6) that was assigned an RBV of 100%. Linearity of the regression curves was determined separately for each diet and a multiple regression model was used to determine the slopes of the test and control iron sources. The RBVs were calculated using the following equation:

% RBV=[Slope of the test diet(1–5)/Slope of the standard diet(6)]×100

The standard error was calculated using the equation $\frac{1}{q}\;\sqrt{SE^{2}q+R^{2}+SE^{2}}p$ where *q* is the slope of the standard diet, and *p* is the slope of the test diet. The standard errors of *p* and *q* are shown as *SE_p_* and *SE_q_*. The relative bioavailability is *R*.

Solubility analyses were conducted in triplicate on Sources 1–5 while six replicates were performed for particle size and surface area measurements. A least squares analysis of variance was performed on the physical property data. Physical properties were considered a function of the source of the iron and therefore fixed effects, while lot was considered a random effect. Independent linear regression analyses at 95% confidence level were performed on particle size, surface area, and moisture uptake measurements *versus* solubility and RBV data. The log_10_ surface area and log_10_ solubility data were used to give a better fit to the data because of the rapid increase in these measurements with increasing amorphous content. Estimates of amorphous content (by DVS moisture uptake) were based on a minimum of two isotherm analyses per source of FePO_4_.

## 3. Results

### 3.1. AOAC Rat Hemoglobin Repletion Bioassay

The iron content of the repletion diets and the change in hemoglobin data are shown in Table 1. Data from the ICP analysis of the iron content of the sources were used to calculate how much of each source to add to the repletion diets. The iron contents of FePO_4_ ranged from 26.4% to 29.3% depending on the number of water molecules of hydration (Mean iron contents, *n* = 2: Source 1, 27.4%; Source 2, 28.4%; Source 3, 28.7%; Source 4, 28.4%; Source 5, 28.1%). All diets contained 4 mg of naturally occurring iron from the malted barley per kg of the cereal formulation. Thus, the endogenous iron added about 4 mg of iron to the total iron content of each diet (4 mg above the target iron dose).

A linearity test was performed on Diets 1–5 comparing the test iron content of each diet *versus* the change in hemoglobin in rats fed each diet during the repletion study. Significant lack-of-fit occurred at *p* ≤ 0.05 when the highest test dose (*i.e.*, 48 mg iron/kg diet; Diets 1–6 mean gain HgB g/L: 69 ± 8; 56 ± 8; 33 ± 9; 64 ± 5; 43 ± 8; 62 ± 9, respectively) for each diet was included in the regression analysis. When the highest iron dose was excluded, linearity was achieved for all diets. As a result, statistical analysis was performed on the data minus the highest dose. Diet 6 contained the ferrous sulfate and was assigned a bioavailability of 100%. RBVs for FePO_4_ sources 1–5 ranged from 51% to 99% (Table 2).

### 3.2. In Vitro Solubility

Solubility results for the iron sources are shown in Table 2. As the acidity of the solutions increased, the solubility of Source 1 increased approximately 50-fold while the solubility of Sources 2–5 increased from two to eight-fold. The iron in Source 1 (2 lots) was the most soluble in 0.1N HCl (18%–21%) followed by Source 4 (1 lot) (1.0%) >Source 2 (1 lot) (0.3%) >Source 3 (2 lots) and Source 5 (1 lot) (0.03%–0.04% and 0.02%, respectively). A regression analysis showed a statistically significant linear relationship between RBV and log_10_ solubility (*R^2^ =* 0.93, *p* = 0.008).

### 3.3. Particle Size and Surface Area

Particle size (D_10_, D_50_ and D_90_) and surface area results are shown in Table 3. The D_10_ statistic measures the smallest particle size population present in a distribution. Data for this value found a significant difference only between Source 1 (10% of the distribution <1.9 ± 0.1 μm) and Source 5 (10% of the distribution <10.4 ± 0.0 μm) (*p* < 0.05). The D_50_ or median particle size data grouped into three statistically different size distributions (*p* < 0.05). Sources 3 and 5 had the highest number of large particles (median particle size 17.7 and 15.5 μm, respectively), followed by Source 1 (median particle size 9.3 μm), while Sources 2 and 4 had the highest number of small particles (median particle size 2 μm for both sources). D_10_ and D_50_ (median) particle size distribution data had the best, though poor, inverse correlation to solubility of the particle size statistics (*R^2^* = 0.38; *p* = 0.10 and *R^2^* = 0.59; *p* = 0.12, respectively) and RBV (*R^2^* = 0.67, *p* = 0.09, and *R^2^* = 0.44, *p* = 0.22 respectively).

Surface area measurements separated the five sources into two significantly different groups made up of Sources 3 and 5 and Sources 1, 2, and 4 (*p* < 0.05). As expected, the sources of FePO_4_ with the largest particles (Sources 3 and 5) had the lowest surface areas (2.0 ± 1.1 and 0.75 ± 0.02 m^2^/g, respectively). Conversely, Sources 1, 2, and 4 were composed of smaller particles than Sources 3 and 5 and had higher surface areas (14.9 ± 0.44, 15.6 ± 2.0, and 11.0 ± 0.1 m^2^/g, respectively). A linear regression analysis of surface area *versus* RBV yielded an *R^2^* value of 0.83 (*p* = 0.03).

### 3.4. Amorphous Content

**Figure 1:**
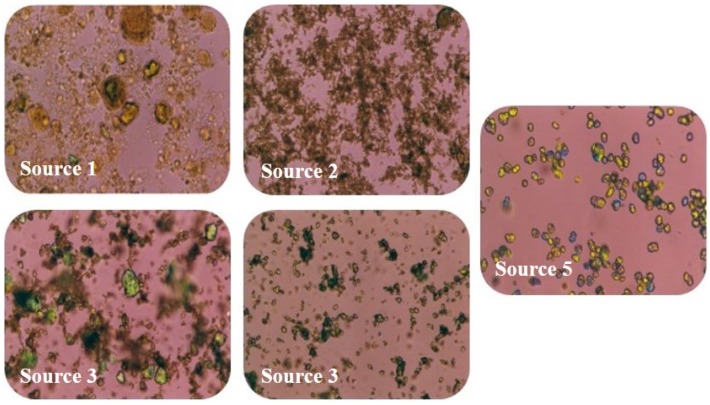
Polarized light photomicrographs (360×) of the FePO_4_ Sources 1–5 showing varying particle size distributions and amounts of crystalline and amorphous material. Highly structured crystalline regions are birefringent and appear brightly colored. Source 1 appears to be almost completely amorphous, containing only a trace of birefringent crystalline structure in some of the larger particles. Source 5 appears to be highly crystalline with most of the particles showing birefringence. Sources 2, 3, and 4 contain mixed amounts of amorphous and crystalline material, based on observations of birefringence.

The microstructure of the FePO_4_ sources was initially investigated using polarized light microscopy. The sources contained differing amounts of amorphous and crystalline material as shown in Figure 1.

**Figure 2:**
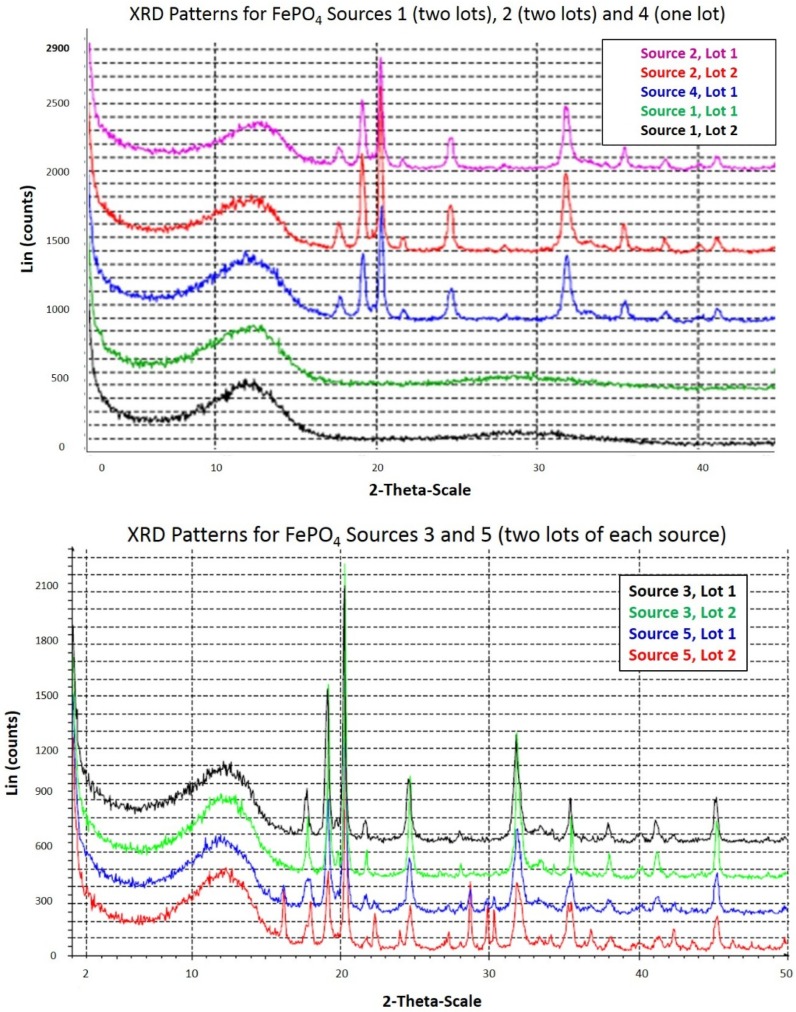
The X-ray diffraction patterns for the five sources of FePO_4_ are displayed from 2 to 50 degrees 2-Theta. The broad peak in the patterns located between 7 and 15 degrees 2 theta represents the amorphous component of the samples. The sharp peaks between 15 and 50 degrees represent crystalline material.

The presence of amorphous material was confirmed qualitatively using X-ray diffraction. X-ray diffraction results are shown stacked for comparison (Figure 2). The X-ray diffraction patterns show that all the Sources, except for Source 1 that was amorphous, contained mixed amounts of amorphous and crystalline material.

The amorphous contents of the samples were also estimated by comparison of moisture uptake by dynamic vapor sorption to the 100% amorphous standard (Table 4). The amorphous contents of Sources 1–5 varied from 1.7% to 23.8% (1% to 14.3% moisture uptake). Source 1 contained approximately 4–10-times the amorphous content of the other sources, depending on the lot tested. Source 2 and Source 4 had the next highest levels of amorphous material followed by Source 3, which had slightly lower amounts. Source 5 had the lowest amorphous content of the samples tested. A linear regression analysis of log_10_ amorphous content *versus* RBV resulted in a *R^2^* = 0.82; *p* = 0.033.

## 4. Discussion

The FePO_4_ sources analyzed in our study exhibited variable RBV consistent with the variability reported in the literature [3,4,5,6]. In two previous rat hemoglobin repletion studies, researchers also added FePO_4_ to infant cereals and RTE breakfast cereals prior to processing. In the first, Rees and Monsen (1973) [9] added FePO_4_ from a single source to a mixed grain infant cereal before cooking and drum drying that resulted in an RBV of 68%. In the second, Shah and coworkers (1979) [19] reported RBVs for two different FePO_4_ fortified breakfast cereals of 33% and at 60%. The RBVs reported in these two studies (RBV 68% and 60%) were higher than typically reported in the literature (RBV 6%–46% (rat); 25%–32% (human)) and comparable to the lower end of the range of RBVs (51% to 99%) found in the current study [1,7]. Neither of these studies characterized the physical or chemical properties of the FePO_4_ sources used making it difficult to draw conclusions about differences between the sources of FePO_4_ and the factors influencing bioavailability.

In this research, the solubility of FePO_4_ in 0.1N HCl was the parameter that correlated most highly with its bioavailability (log_10_ solubility, *R^2^* = 0.93, *p* = 0.008). The log_10_ transformation was used because of the very high solubility of Source 1 compared to the other sources. This finding is consistent with the SUSTAIN Task Force study in which the solubility of elemental iron in 0.1N HCl was also found to be highly predictive of its RBV (*R^2^* = 0.86, *p* < 0.01) [10]. Coccodrilli and coworkers (1976) [8] also found a positive relationship between the solubility of four commercial sources of FePO_4_ in 10% HCl (5.8%–95.6%) and their corresponding RBV (4%–44%) in rat hemoglobin repletion studies (statistical correlation was not provided). An important determinant of solubility is the surface area of the particles. A linear regression of surface area data for FePO_4_ *versus* log_10_ solubility showed a positive relationship (*R^2^* = 0.83; *p* = 0.002). Decreasing particle size is often the approach taken to increase the surface area of materials; therefore, the particle size of poorly soluble forms of iron such as elemental iron [10], ferric pyrophosphate [20,21,22], and ferric/ferrous ammonium phosphate is decreased to increase dissolution rate [13,23]. In the SUSTAIN study, the surface area of elemental iron powders was also predictive of RBV (*R^2^* = 0.81, *p* < 0.01). Median (D_50_) particle size of elemental iron had a significant inverse relationship to RBV but was less well correlated with RBV (*R^2^* = 0.64, *p* < 0.01) than surface area. The findings for elemental iron are consistent with the results for FePO_4_ in this study in which the surface area of FePO_4_ was a better predictor of RBV (*R^2^* = 0.83; *p* = 0.03) than its median (D_50_) particle size (*R^2^* = 0.43, *p* = 0.22) or D_10_ particle size (*R^2^* = 0.67, *p* = 0.09). The weaker correlation of particle size *versus* surface area to RBV suggests that another factor is contributing to the solubility of FePO_4_ and ultimately its bioavailability. Particle size measurements performed by light scattering and sieve techniques do not measure the internal porosity of particles and neither particle size nor surface area measurements characterize their crystalline structure.

Highly organized crystalline material can have varying degrees of molecular disorder, referred to as amorphous content, within its structure [24,25]. These amorphous regions are in a higher energy state and are more thermodynamically unstable than their crystalline counterparts; amorphous regions exhibit a higher absorption of water vapor that will result in increased solubility and reactivity [24]. FePO_4_ is a semi-crystalline compound with varying degrees of molecular disorder within its particle structure, depending on its method of manufacture. Amorphous material can be formed during crystallization and subsequent processing and is often located on the particle surface increasing surface reactivity [24]. Amorphous FePO_4_ has been shown to be an important determinant of bioavailability for plants and insects, and is hypothesized to be an important factor for its efficacy in humans [25]. Amorphous FePO_4_ is extremely hydroscopic, which allows it to be quantified by DVS isotherms that measure the change in mass due to absorbed moisture, or moisture uptake. DVS analysis is a very sensitive technique that is commonly used in the pharmaceutical industry to measure the presence of small amounts of amorphous material in drug preparations [26,27]. It was applied in this research to quantify the amorphous content of FePO_4_.

The amorphous content of the FePO_4_ sources by DVS analysis ranged from 1.7% to 23.8%. Using the log_10_ of both the amorphous content and solubility measurements, amorphous content was a better predictor of solubility (*R^2^* = 0.91; *p* = 0.0002) than median D_50_ particle size (*R^2^* = 0.59, *p* = 0.12) and was highly correlated to RBV (*R^2^* = 0.82; *p* = 0.03). Source 1 had the highest RBV (99%), highest solubility in 0.1N HCl (19.5%, average of two lots), and highest amorphous content (23.8%) of the five sources tested. Sources 2 and 4 had intermediate RBV (78% and 83%, respectively), solubility in 0.1N HCl (0.3%, 1.0%, respectively) and amorphous contents (5.3% and 5.7%, respectively). Sources 3 and 5 had the lowest RBV (51% and 60%, respectively), solubility in 0.1N HCl <0.1%, and amorphous contents (3.0% and 2.0%, respectively). Several lots of FePO_4_ were available from selected FePO_4_ sources for analysis and there were modest differences in physiochemical properties from lot to lot, but these lot to lot differences did not overlap the source to source differences. The use of *in vitro* predictive physiochemical screening tests of various lots of FePO_4_ from a single source would help assure consistent bioavailability.

Two previous bioavailability studies investigated the amorphous state of FePO_4_ particles in addition to measuring its solubility in dilute HCl. In the first study, Hallberg and coworkers (1989) [13] prepared crystalline and amorphous FePO_4_. The two forms of FePO_4_ were added to flour that was baked into rolls and fed to human test subjects in a series of bioavailability studies. No analytical information was provided that characterized the amorphous or crystalline state of the FePO_4_ particles or measured the amount of amorphous material present. Results showed that the amorphous FePO_4_ had a higher RBV than crystalline FePO_4_ (RBV 33% and 10%, respectively) and was over five-times more soluble (pH 1.0 HCl) than its crystalline counterpart [13]. In the second study, Rohner and coworkers (2007) [20] compared the relative bioavailability of a commercially available FePO_4_ powder to two experimentally produced amorphous, nano-sized FePO_4_ powders using the AOAC Rat Hemoglobin Repletion Bioassay. They measured the solubility of the sources (pH 1.0 HCl as compared to ferrous sulfate) and characterized the morphology and amorphous state by transmission electron microscopy/selected area electron diffraction, surface area by BET, and particle size by laser light scattering. The two nano-sized samples (median particle sizes of 30.5 and 10.7 nm) had solubility relative to ferrous sulfate of 79% and 85%, surface areas of 68.6 and 194.7 m^2^/g, and RBV of 73% and 96%, respectively. [20]. Nano-sized materials are intentionally produced materials that have one or more dimensions ≤100 nm. Such materials have increased surface areas and can have chemical, physical, and biological properties that differ from those of their larger counterparts [28]. The commercial FePO_4_ was made up of large, amorphous, and irregularly shaped porous particles that had 73% of the solubility of ferrous sulfate, a median particle size of 2.5 μm; surface area 32.6 m^2^/g; and RBV 61%. The authors noted that in addition to particle size, RBV may have been influenced by amorphous state and internal porosity contributing to high surface area (although these factors were not controlled as part of their study).

To the authors’ knowledge, this research is the first to use dynamic vapor sorption to quantify the amount of amorphous material contained in a range of well characterized, food-grade, commercial FePO_4_ sources in which *in vivo* RBV was determined by the AOAC Rat Hemoglobin Repletion Bioassay using the same lots of FePO_4_. The rat model is not the ideal predictor of bioavailability in humans since the physiology of iron absorption in rats is different than in humans. Rats are not as affected by substances in the diet that interfere with iron absorption such as phytic acid because rats in contrast to humans have the ability to produce phytase which degrades dietary sources of phytic acid [1,29,30,31]. Rats in contrast to humans are also able to synthesize ascorbic acid in their liver and secrete it into their gastric juice [32]. Ascorbic acid is known to increase the bioavailability of iron in foods [1,29,31,33]. RTE-cereals can be high in phytate, containing from 0.05% to 3.29%, depending on the type of grain and portion of the grain used. However, the phytate content of the RTE-cereal used to make the rat repletion diets was low (0.05%–0.09%) because it was made from corn grits which have had the germ and outer layers of the grain removed. These components of the corn kernel contain the highest amounts of phytate [30,34]. In addition, the RTE-cereal was fortified with 0.2 mg/g ascorbic acid that may have contributed to higher than expected RBVs. Although the rat hemoglobin repletion bioassay is not an ideal predictor of human bioavailability, it can provide accurate relative estimates of bioavailability when comparing different iron sources and samples within a single source.

Several researchers have compared the efficacy of the AOAC Rat Hemoglobin Repletion Bioassay with human bioavailability methods. Forbes and others (1989) [18] compared the human radioisotope extrinsic tag technique with the AOAC Rat Hemoglobin Repletion Bioassay. They concluded that the AOAC rat model was the economical method of choice for predicting relative bioavailability in humans. In the Sustain Task Force study, nine commercial elemental iron powders were evaluated by screening approaches to predict bioavailability in humans, including solubility in 0.1N HC and the AOAC Rat Hemoglobin Repletion Bioassay. The predicted RBVs were compared with the RBVs obtained from the human plasma iron tolerance test. The authors concluded that, “human plasma iron tolerance tests were in general agreement with the other measures of predicted bioavailability but they did not provide information that would have improved the precision of bioavailability estimates based on physical properties, dissolution in HCl and/or RBV in rats” [10].

Results of our research found that solubility gave the best prediction of RBV and is promising as an *in vitro* tool for predicting the bioavailability FePO_4_. While solubility is highly predictive of RBV, solubility, in turn, is strongly linked to surface area and amorphous content. Amorphous FePO_4_ was shown to be extremely hygroscopic by dynamic vapor sorption measurements, indicating it plays a key role in solubility. Dynamic vapor sorption is a sensitive technique to measure amorphous content and can potentially be used at a process control point to consistently produce FePO_4_ with desirable bioavailability. The fortification of foods with iron requires balancing the benefits of using highly bioavailable iron with concerns for the pro-oxidant and negative organoleptic effects on product quality. RTE breakfast cereals are especially difficult to fortify with iron because their polyunsaturated lipid content makes them prone to oxidative rancidity, especially given the harsh cereal processing conditions such as pressure cooking, high shear milling, and extrusion processes. Highly soluble forms of iron such as ferrous sulfate and ferrous fumarate are unsuitable from a food quality perspective for many sensitive food applications; thus, affordable, nutritionally acceptable alternatives are needed. Based on 2016 market pricing and considering the iron content of the different iron sources, FePO_4_ (29% iron) is three-times more expensive than ferrous sulfate heptahydrate (20% iron), 2.3-times more expensive than ferrous fumarate (32%), and about 1.5-times more expensive than food-grade ferric pyrophosphate (21% iron) [35]. Because data on the bioavailability of FePO_4_ is limited, variable and often poor, its use is not permitted in the European Union [36] and is limited elsewhere. This keeps production volumes low and increases the cost of this fortificant. Variable stability and organoleptic differences among the commercially available FePO_4_ powders also limit its use. The distinct quality differences between the FePO_4_ powders used in this research carried through to the RTE-cereal. The rat diets made with the poorest FePO_4_ source from an organoleptic point of view had the highest solubility, amorphous content, and RBV of the sources in the study. This was true of the other FePO_4_ sources in which product quality decreased as solubility, amorphous content and RBV increased. Cereals made with Source 1 (and ferrous sulfate) in particular had gray-green discoloration and a metallic off-flavor. Fortification with Source 5 resulted in excellent quality product without defects.

In conclusion, it is possible to select FePO_4_ sources with acceptable to good organoleptic properties that have consistent and desirable RBV in RTE-cereal applications. Future research is needed to further investigate the relationship between amorphous content and the bioavailability of FePO_4_ powders with amorphous contents between 5% and 25%. Dynamic vapor sorption has potential as a new screening tool to measure amorphous content in FePO_4_ powders to predict solubility and produce powders with consistent RBV and satisfactory organoleptic properties.

## Figures and Tables

**Table 1:** Iron Content of the Repletion Diets and Hemoglobin Gain of the Rats ^a^ Fed the Diet.

Target Test Iron Dose ^b^ (mg of Iron/kg Diet) ^c^			
Test Iron and Hemoglobin Gain	6 mg/kg	12 mg/kg	24 mg/kg
Diet 1, Source 1 (mg iron/kg diet)	11	17	27
Hemoglobin gain (g/L)	−3 ± 6	16 ± 6	53 ± 6
Diet 2, Source 2 (mg iron/kg diet)	11	16	28
Hemoglobin gain (g/L)	−5 ± 3	6 ± 5	40 ± 6
Diet 3, Source 3 (mg iron/kg diet)	11	16	29
Hemoglobin gain (g/L)	−10 ± 4	−1 ± 6	22 ± 9
Diet 4, Source 4 (mg iron/kg diet)	10	16	28
Hemoglobin gain (g/L)	−5 ± 4	9 ± 7	40 ± 5
Diet 5, Source 5 (mg iron/kg diet)	10	14	25
Hemoglobin gain (g/L)	−10 ± 3	−3 ± 5	20 ± 6
Diet 6, Source 6 ferrous sulfate (mg iron/kg diet)	10 ± 1	16 ± 2	25 ± 1
Hemoglobin gain (g/L)	−2 ± 9	22 ± 11	44 ± 11

^a^ Mean ± SD of 10 animals per study group per FePO_4_ Source tested; ^b^ Hemoglobin values at the zero dose level (no added FePO_4_) were −21 g/L ± 5; ^c^ Diets 1–5, all dose levels (*n* = 1); Diet 6 standard ferrous sulfate: dose level 0 mg/kg (*n* = 2), dose levels 4 and 6 mg/kg (*n* = 4), dose level 24 mg/kg (*n* = 5).

**Table 2:** Percent RBVs and Solubility of Iron for the Five FePO_4_ Sources.

FePO_4_ Source		Iron Dissolved (mg)/Total Iron (g) ^a,b^		
	%RBV ± SD ^c,d^	0.02N HCl	0.05N HCl	0.1N HCl
Source 1				
lot 1 (*n* = 1)	99 ± 9 a	4.44	41.44	211.0
lot 2 (*n* = 3)	‒	8.13 ± 0.06	49.43 ± 0.21	179.0 ± 1.0
Source 2				
lot 1 (*n* = 3)	78 ± 7 b	0.65 ± 0.21	1.36 ± 0.19	2.88 ± 0.21
Source 3				
lot 1 (*n* = 3	51 ± 5 d	0.12 ± 0.009	0.23 ± 0.02	0.29 ± 0.02
lot 2 (*n* = 3)	‒	0.16 ± 0.005	0.23 ± 0.01	0.40 ± 0.07
lot 3 (*n* = 2)	‒	0.10 ± 0.002	0.15 ± 0.05	0.33 ± 0.04
Source 4				
lot 1 (*n* = 3)	83 ± 7 b	1.28 ± 0.03	4.20 ± 0.03	10.09 ± 0.12
Source 5				
lot 1 (*n* = 2)	60 ± 6 c	0.10 (*n* = 1)	0.10 ± 0.01	0.23 ± 0.01
pooled variance	‒	8.588	13.676	176.982
pooled SD	‒	0.093	0.117	0.421
pooled assay CV	‒	5.00%	1.10%	0.88%

^a^ Solubility of iron in FePO_4_ was calculated as mg iron dissolved (by ICP analysis) per g of iron analyzed in each concentration of acid after the dissolution period followed by filtration of remaining undissolved iron; ^b^ Values are the Mean ± SD. *n* = number of replicate analyses per lot; ^c^ Mean ± SD of 10 animals per each test diet; ^d^ RBVs with different letters (a,b,c,d) are significantly different (*p* < 0.05); standard ferrous sulfate (not shown) is 100% soluble and assigned a %RBV of 100.

**Table 3:** Particle Size and Surface Area of FePO_4_ sources ^a,b^.

FePO_4_ Sources	Mean	D_10_ (μm)	Median D_50_ (μm)	D_90_ (μm)	Surface Area (m^2^/g)
Source 1					
lot 1	21.5 ± 1.1	1.9 ± 0.1	9.3 ± 0.4	59.0 ± 2.1	14.4 ± 0.0
lot 2	20.4 ± 1.2	2.0 ± 0.1	9.2 ± 0.6	56.3 ± 3.2	15.3 ± 0.1
Mean	21.0 ± 1.2 a	1.9 ± 0.1 a	9.3 ± 0.4 a	57.6 ± 2.9 a	14.9 ± 0.44 a
Source 2					
lot 1	7.2 ± 0.2	0.9 ± 0.1	2.2 ± 0.0	22.0 ± 0.9	13.7± 0.2
lot 2	4.0 ± 0.4	0.9 ± 0.1	1.8 ± 0.0	11.0 ± 0.6	17.6 ± 0.3
Mean	5.6 ± 1.6 b	0.9 ± 0.1 a,b	2.0 ± 0.2 b	16.5 ± 5.8 b	15.6± 2.0 a,b
Source 3					
lot 1	18.6 ± 0.2	8.2 ± 0.2	17.1 ± 0.1	31.9 ± 0.4	1.5 ± 0.0
lot 2	20.4 ± 0.1	3.9 ± 0.1	19.4 ± 0.1	35.6 ± 1.6	1.1 ± 0.0
lot 3	18.0 ± 0.2	5.4 ± 0.3	16.6 ± 0.1	32.5 ± 0.3	3.6 ± 0.2
Mean	19.0 ± 1.0 a,c	5.8 ± 1.9 a,b,c	17.7 ± 1.3 c	33.5 ± 1.9 b,c	2.0 ± 1.1 c
Source 4					
lot 1	5.4 ± 0.2 b	0.8 ± 0.0 a,b,c	2.0 ± 0.0 b	15.9 ± 0.9 b,c,d	11.0 ± 0.1 a,b
Source 5					
lot 1	16.3 ± 0.0 a,c	10.4 ± 0.0 b	15.5 ± 0.0 c	23.3 ± 0.1 b,c,d	0.75 ± 0.02 c

^a^ Values represent the mean ± SD of 6 measurements for particle size and surface area; ^b^ Means within a column without letters (a,b,c,d) in common are significantly different (*p* < 0.05).

**Table 4:** Amorphous Content of FePO_4_ Sources.

Source	Moisture Uptake ^a^ (%)	Amorphous Content (%)
Source 1		
lot 1	14.3	23.8
lot 2	9.3	15.5
Source 2		
lot 1	3.2	5.3
lot 2	3.9	6.5
Source 3		
lot 1	1.8	3.0
lot 2	1.0	1.7
lot 3	2.5	4.2
Source 4		
lot 1	3.4	5.7
Source 5		
lot 1	1.2	2.0

^a^ Moisture uptake is the mass increase due to moisture gain during DVS analysis.
